# Calcium-controlled conformational choreography in the N-terminal half of adseverin

**DOI:** 10.1038/ncomms9254

**Published:** 2015-09-14

**Authors:** Sakesit Chumnarnsilpa, Robert C. Robinson, Jonathan M. Grimes, Cedric Leyrat

**Affiliations:** 1 Division of Structural Biology, University of Oxford, Henry Wellcome Building for Genomic Medicine, Oxford OX3 7BN, UK; 2 Institute of Molecular and Cell Biology, A*STAR (Agency for Science, Technology and Research), Biopolis, Singapore 138673, Singapore; 3 School of Biochemistry, Institute of Science, Suranaree University of Technology, 111 University Avenue, Muang, Nakhon Ratchasima 30000, Thailand; 4 Department of Biochemistry, National University of Singapore, 8 Medical Drive, Singapore 117597, Singapore; 5 Science Division, Diamond Light Source Ltd, Diamond House, Harwell Science and Innovation Campus, Didcot, Oxfordshire OX11 0DE, UK

## Abstract

Adseverin is a member of the calcium-regulated gelsolin superfamily of actin-binding proteins. Here we report the crystal structure of the calcium-free N-terminal half of adseverin (iA1–A3) and the Ca^2+^-bound structure of A3, which reveal structural similarities and differences with gelsolin. Solution small-angle X-ray scattering combined with ensemble optimization revealed a dynamic Ca^2+^-dependent equilibrium between inactive, intermediate and active conformations. Increasing calcium concentrations progressively shift this equilibrium from a main population of inactive conformation to the active form. Molecular dynamics simulations of iA1–A3 provided insights into Ca^2+^-induced destabilization, implicating a critical role for the A2 type II calcium-binding site and the A2A3 linker in the activation process. Finally, mutations that disrupt the A1/A3 interface increase Ca^2+^-independent F-actin severing by A1–A3, albeit at a lower efficiency than observed for gelsolin domains G1–G3. Together, these data address the calcium dependency of A1–A3 activity in relation to the calcium-independent activity of G1–G3.

## Abstract (web summary)

The calcium-regulated gelsolin family of actin-binding proteins includes adseverin. Here, the authors report the X-ray crystal structure of the N-terminal portion of adseverin and use small-angle scattering and molecular dynamics simulations to examine the calcium-dependent function of this protein.

## Graphical abstract

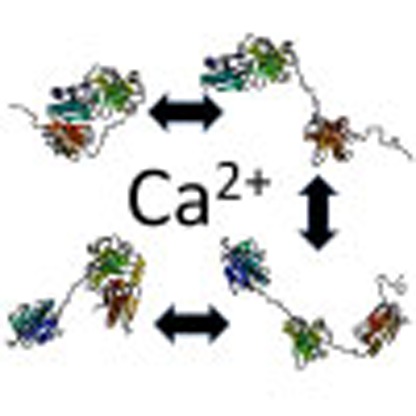

Adseverin (also known as scinderin) is a member of the gelsolin superfamily of Ca^2+^-dependent actin-binding proteins, which regulate cytoskeletal dynamics by severing, bundling and capping of actin filaments^1^. The gelsolin superfamily groups a number of modular, evolutionary-related proteins that are composed of 3–6 globular gelsolin homology (GH) domains often fused to non-GH N- and/or C-terminal extensions^2^. In humans, family members include gelsolin, adseverin, villin, supervillin, advillin, villin-like protein, flightless I and CapG (reviewed in ref. 3). Adseverin was first identified in chromaffin cells of bovine adrenal medulla, where it acts as an actin depolymerizing agent that disassembles the cortical network of actin filaments in response to bursts of intracellular calcium, leading to exocytosis of secretary vesicles^4^,^5^,^6^. Adseverin has also been shown to be involved in the regulation of osteoclastogenesis^7^ and thrombocytic^8^, chondrocytic^9^ and odontoblastic differentiation^10^, as well as in cisplatin resistance in bladder cancer^11^. Recently, several studies revealed a role for adseverin in the proliferation of lung carcinoma^12^, and gastric^13^ and prostate cancer cell lines^14^.

Adseverin is composed of six GH domains (A1–A6) and shares 60% overall sequence identity with gelsolin (G1–G6). Adseverin and gelsolin have been shown to have two calcium-binding events, with Kds of 0.6 and 3 μM for adseverin^4^, and Kds of 0.3 and 1.2 μM for gelsolin^15^. Low-affinity calcium association occurs with Kds of 25 and 200 μM for gelsolin^16^. Crystallographic studies revealed that these binding events involve the association of eight calcium ions, six of which bind at a conserved site on each domain (type II), whereas the remaining two are also stabilized by contacts with actin (type I; refs 17, 18, 19, 20). Both proteins are able to sequester two monomers of actin, as well as sever and cap actin filaments through distinct Ca^2+^-induced domain rearrangements of the two halves of the molecule^4^,^17^,^18^,^21^,^22^,^23^. In addition, both proteins are inhibited by membrane lipids such as PIP and PIP_2_, although adseverin is additionally rendered inactive by phosphatidylinositol and phosphatidylserine^24^,^25^.

The crystal structure of calcium-free gelsolin^26^ revealed an inactive, globular state, where all the actin-binding sites are obscured by three Ca^2+^-dependent latches. In this state, the two halves of the molecule interact and adopt a pseudo-symmetric conformation in which the major actin-binding sites on domains G1 and G4 are, respectively, buried by interactions with G3 and G6, while the actin-binding site on G2 is masked by interactions with the C-terminal α-helical extension, which is absent in adseverin. In this conformation, the long α-helices of G3 and G6 adopt a kinked structure, which is straightened by the Ca^2+^-induced release of the latches^27^.

The structures of the N- and C-terminal halves of gelsolin in complex with actin^17^,^18^ have demonstrated that distinct Ca^2+^-induced domain rearrangements of each half of the molecule are crucial for actin binding. The N-terminal half adopts a conformation in which a new Ca^2+^-stabilized G2:G3 interface is formed while G1 dissociates from the rest of the molecule to bind in the groove between actin subdomains 1 and 3 (refs 18, 20). In contrast, the C-terminal half adopts a compact V-shaped structure where the G4/G6 latch is released by flipping over G6 to create a new G5:G6 interface^17^,^22^. Small-angle X-ray scattering (SAXS) studies showed a significant calcium-induced increase in the molecular dimensions of gelsolin, and *ab initio* reconstructions suggested an opening of the two halves and domain rearrangements consistent with the architecture of the active forms observed crystallographically^28^.

Despite extensive homology between adseverin and gelsolin, adseverin lacks the gelsolin C-terminal α-helix latch, a feature that likely explains a less complex relationship between severing rate and calcium concentration than observed for gelsolin^21^. In addition, the actin filament severing and capping activities of the N-terminal half of adseverin (A1–A3) are Ca^2+^ dependent^29^, whereas the same activities of G1–G3 are Ca^2+^ independent^30^. These differences, combined with similar activated structures and actin sequestering properties of the C-terminal halves, led to the proposal that the activation process of adseverin is dominated by calcium binding to its N-terminal half^23^. Recently, SAXS studies demonstrated that G1–G3 adopts an active conformation in the absence of calcium^31^, suggesting that the Ca^2+^-independent severing activity of this fragment results from instability of the G1/G3 latch in the absence of interaction with G4–G6 and the C-terminal α-helix latch.

Here we investigated the structure, dynamics and calcium activation of A1–A3 by using a combination of X-ray crystallography, SAXS, molecular dynamics simulations (MDS) and ensemble optimization. We crystallized A1–A3 in the presence of EGTA, revealing a compact auto-inhibited structure similar to the conformational state observed in inactive full-length gelsolin. Unexpectedly, attempts to crystallize the active form of A1–A3 in the presence of 5 mM calcium led to a structure of Ca^2+^-bound A3 following protein degradation within the crystallization drop. Using SAXS-based atomic ensemble optimization, we were able to draw a comprehensive picture of the conformational landscape of A1–A3 and track Ca^2+^-induced conformational changes. Classical MDS provided insights into the calcium-induced destabilization of inactive A1–A3, and revealed the location of potential low-affinity calcium-binding sites. Finally, actin depolymerization assays showed that mutations that destabilize the inactive A1:A3 interface increase A1–A3 severing activity in calcium-free conditions, demonstrating the importance of this latch.

## Results

### Calcium-free A1–A3 displays an inactive conformation

**Figure 1:**
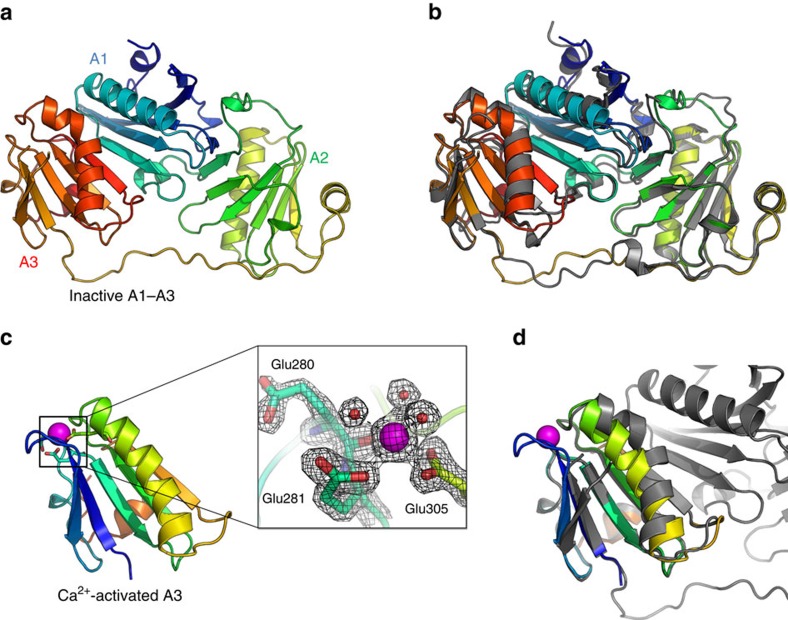
Crystal structures of the N-terminal half of adseverin in calcium-free conditions (iA1–A3) and of calcium-bound domain A3 (aA3). (**a**) The structure of iA1–A3 (residues 6σ349) is shown in cartoon representation and coloured from blue to red (N- to C terminus). (**b**) Comparison of the structure of iA1–A3 with domains iG1–G3 of gelsolin excised from the structure of full-length gelsolin in calcium-free conditions (PDB 1D0N)^26^, showing a similar compact arrangement of the domains that masks the conserved actin-binding interfaces on A1 and A2. G1–G3 is shown in grey cartoon representation. (**c**) The calcium-bound structure of adseverin domain A3 (aA3, residues 248–349), shown in cartoon representation and coloured from blue to red (N- to C terminus). The calcium ion is shown as a magenta sphere with coordinating residues as sticks. Inset: close up of the calcium-binding site, showing the coordination of the calcium ion by the Glu280 carbonyl, monodentate Glu281 and bidentate Glu305 side chains, as well as three water molecules. The 2*F*_o_*-F*_c_ electron density map contoured at 1.5*σ* is shown as a mesh. (**d**) Comparison of the structure of aA3 (coloured as blue to red rainbow) and iA1–A3 (in grey). Superimposition highlights the conformational changes in the second β-strand and the long α-helix of A3 in response to calcium binding and dissociation from A1.

Purified recombinant A1–A3, supplemented with 5 mM EGTA, crystallized in space group C2 and its structure was solved by molecular replacement using the structure of G1–G3 excised from inactive full-length gelsolin as the search model and refined to a resolution of 2.9 Å (Fig. 1a and Table 1). Each domain adopts the classic GH fold, which is organized around a central five-stranded mixed β-sheet sandwiched between a long α-helix running approximately parallel to the strands, and a shorter α-helix running approximately perpendicular to the strands. The domains adopt the compact arrangement characteristic of inactive gelsolin, with A1 sandwiched between A2 and A3. The structure appears to be relatively rigid, with B-factors indicating that flexibility is limited mainly to the extended A2A3 linker and the short α-helices of A1 and A2 (Supplementary Fig. 1a). In this inactive conformation, the long α-helix of A3 is kinked to enable the formation of the A1/A3 latch, resulting in the masking of the major actin-binding interface on A1. The inactive forms of A1–A3 (iA1–A3) and G1–G3 (iG1–G3) are highly similar with an overall root mean squared deviation (r.m.s.d.) in Cα positions of 1.19 Å over 337 aligned residues, highlighting the structural conservation between gelsolin and adseverin (Fig. 1b). It is noteworthy that contrary to adseverin, iG1–G3 is unstable in the absence of G4–G6, leading to an open conformation of G1–G3 in the presence of EGTA^31^. The iA1–A3 crystal lattice is stabilized by a number of crystal contacts, including a relatively large packing interface of ∼1,518 Å^2^ (Supplementary Fig. 1b). Interestingly, comparisons with full-length gelsolin show that this interface largely buries the surfaces in iA1–A3 that bind the C-terminal half of the molecule, suggesting that the crystal environment partially mimics the inactive full-length protein (Supplementary Fig. 1c).

### Calcium-bound structure of A3

Attempts to crystallize the active form of A1–A3 (aA1–A3) using a 5-mM CaCl_2_ supplemented protein sample led to the appearance of a single crystal after ∼80 days, from which a 1.8-Å data set was collected. Cell content analysis indicated that the asymmetric unit was too small to accommodate more than a single GH domain, and the structure was solved by molecular replacement using gelsolin domain G3 excised from the structure of actin-bound G1–G3 as a search model (aG3) (PDB 1RGI). This finding was surprising given that homology with gelsolin suggests that in the active form, domains A2 and A3 interact through a Ca^2+^-stabilized interface, while A1 would be separated by the A1A2 linker^18^,^20^; it would thus have been expected that degradation would lead to a structure of isolated A1, or a compact, Ca^2+^-stabilized structure of A2–A3, rather than the Ca^2+^-bound A3 structure obtained here.

The conformation of aA3 is highly similar to aG3, with a r.m.s.d. in Cα positions of 0.82 Å over 93 Cα atoms, and differs from inactive iA3 and iG3 by the position of the second β-strand and the straightening of the long α-helix (Fig. 1c,d). The structure is stabilized by Ca^2+^ coordination at the type II binding site, which involves interactions with Glu280 carbonyl, the carboxylates of Glu281 and Glu305, and three water molecules with bond distances ranging between 2.3 and 2.5 Å (Fig. 1c, inset, and Supplementary Fig. 1d). As previously noted for gelsolin, this Ca^2+^-bound conformation is incompatible with the formation of the A1–A3 latch (Fig. 1d), suggesting that calcium binding at A3 moves A1–A3 away from the inactive conformation through straightening of the A3 long helix resulting in the disruption of the A1:A3 interface^18^,^27^.

Taken together, these results indicate that the Ca^2+^-induced conformational changes in adseverin mirror those observed in gelsolin, although in isolation iA1–A3 retains the inactive conformation that is lost by isolated Ca^2+^-free G1–G3. Furthermore, the structure of aA3 obtained by degradation of A1–A3 in the presence of calcium indicates that A3 may exist in a conformational state where it is distant from A1 and A2.

### SAXS experiments

**Figure 2:**
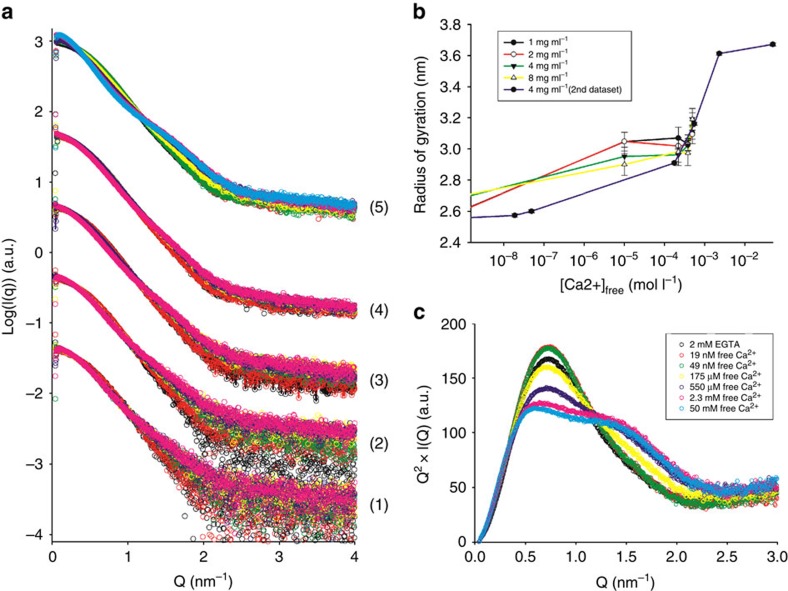
SAXS experiments. (**a**) SAXS profiles of A1–A3 in the presence of 10 mM EGTA (black spheres), 2 mM EGTA (red spheres) and with increasing calcium concentrations (10, 220, 390 and 490 μM CaCl_2_, green, yellow, purple and pink spheres, respectively) measured at protein concentrations of 1, 2, 4 and 8 mg ml^−1^ (curve series 1, 2, 3 and 4, respectively). Curve series (5) corresponds to a second calcium titration at 4 mg ml^−1^ that spans a larger range of calcium concentrations (2 mM EGTA, 19 and 49 nM, 175 and 550 μM and 2.3 and 50 mM free Ca^2+^) and was measured on a separate synchrotron visit using a different protein preparation. (**b**) Changes in radius of gyration as a function of calcium concentration corresponding to the curve series in **a**: (1) black, (2) red, (3) green, (4) yellow and (5) purple line. (**c**) Model-free analysis of calcium-induced conformational changes. The data shown in **a**, curve series (5) is represented as Kratky plots, indicating a transition towards a less globular and more flexible structure.

Next, we investigated the structure and calcium activation of A1–A3 using solution SAXS. Scattering profiles for A1–A3 were initially measured in the presence of 2 or 10 mM EGTA, and with increasing concentrations of CaCl_2_ (10–500 μM of free Ca^2+^), at protein concentrations of 1, 2, 4 and 8 mg ml^−1^ (Fig. 2a, curve series 1–4). The samples were aggregation free, as evidenced by the linearity of the Guinier region (Supplementary Fig. 2), with radii of gyration (*R*_g_) ranging between 2.6 and 3.2 nm, and molecular weight estimates ranging from 35 to 42 kDa (Table 2), consistent with the theoretical value of 41.5 kDa. The Ca^2+^-induced changes in the scattering profiles were more pronounced at lower protein concentrations, which is to be expected as the Kds for the high-affinity calcium-binding sites are lower than the protein concentrations used in the experiment (*K*_d_=0.6 and 3 μM versus *c*=24–192 μM). An additional calcium titration was performed at 4 mg ml^−1^ of protein using an increased range of calcium concentrations, in order to check for potential changes at very low (10 or 50 nM), as well as higher calcium concentrations (2 or 50 mM; Fig. 2a, curve series 5, and Table 2). These measurements, performed on a different synchrotron visit using a different protein preparation, showed Ca^2+^-induced changes that were similar to those observed in the previously acquired data (Fig. 2a, curve series 3, Supplementary Fig. 3a). All measured *R*_g_ were, however, smaller by 1–2 Å, suggesting mild degradation of the sample (Fig. 2b and Table 2). To gain qualitative insights into the Ca^2+^-induced conformational changes, the data were plotted using the Kratky representation (Fig. 2c). The analysis revealed that A1–A3 is predominantly globular in the absence of calcium, resulting in a bell-shaped curve, and that addition of calcium leads to a more complex profile suggesting modularity and significant interdomain flexibility^32^.

### Ca^2+^-dependent activation of A1–A3

**Figure 3:**
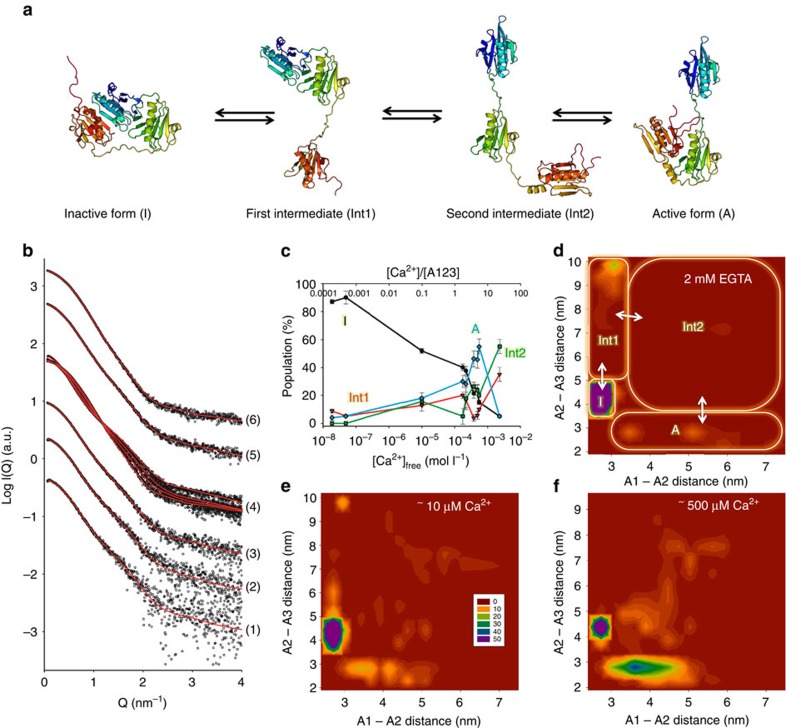
Proposed mechanism of calcium activation of A1–A3 in solution. (**a**) Model for A1–A3 transition between the inactive and active (actin-binding competent) states. The release of the A1/A3 latch from the inactive form (I) leads to a first intermediate (Int1), followed by the loss of the A1A2 interface (Int2). Finally, the calcium-stabilized interface between A2 and A3 is formed, resulting in the active conformation (**a**). (**b**) Fitted SAXS profiles. For clarity, only a subset of the data shown in Fig. 2a is represented. Experimental data are shown as black spheres and theoretical SAXS profiles for optimized ensembles of 50 models are drawn as red lines. (1) 490 μM CaCl_2_, 1 mg ml^−1^ of A1–A3; (2) 490 μM CaCl_2_, 2 mg ml^−1^ of A1–A3; (3) 490 μM CaCl_2_, 4 mg ml^−1^ of A1–A3; (4) 49 nM, 175 and 550 μM, and 2.3 mM CaCl_2_, 4 mg ml^−1^ of A1–A3; (5) 490 μM CaCl_2_, 8 mg ml^−1^ of A1–A3; (6) 2 mM EGTA, 8 mg ml^−1^ of A1–A3 mg ml^−1^. (**c**) Relative populations of inactive (I, black), intermediate (Int1, red and Int2, green) and active (A, blue) conformations of A1–A3, plotted as a function of free calcium concentration (*x* axis, bottom) and ratio of calcium ion over protein (*x* axis, top). All data measured at 4 mg ml^−1^ were used (Fig. 2 A, curve series 3 and 5). Error bars represent the s.d. of ensemble optimization results obtained from 10 independent ensembles of 6,000 models (see Methods section). (**d**–**f**) Two-dimensional (2D) histogram representations of the distribution of A1–A2 and A2–A3 interdomain distances (taken between their centres of mass) within the optimized ensembles, using data measured in the presence of 2 mM EGTA (**d**), 10 μM (**e**) and 550 μM free Ca^2+^ (**f**). The 2D histogram was calculated using a binning of 15 × 15 and coloured from red to blue (0–50 models).

In order to analyse Ca^2+^-induced changes detected by SAXS, we combined atomistic coarse-grained MDS with ensemble optimization (Supplementary Note 1), demonstrating that A1–A3 transitions from the inactive state (I) to a gelsolin-like active state (A) through two distinct intermediates (Int1 and Int2) characterized by the release of A1/A3 latch and subsequent loss of the A1:A2 interface, respectively (Fig. 3a). All available data could be well fitted using this approach (Fig. 3b), with a goodness of fit *χ*_exp_<2 for 0.08<Q<5 nm^−1^ (Table 2), except for data measured in the presence of 50 mM free calcium (*χ*_exp_=2.05), which was not analysed further. Data measured at 4 mg ml^−1^ (Fig. 2a, curve series 5), which showed mild signs of degradation, could, however, only be optimally fitted using ensembles in which the last disordered 15 amino acids were truncated (residues 350–365—part of the A3A4 linker; Table 2 and Supplementary Fig. 4d,e). To understand the nature of Ca^2+^-induced conformational changes, we analysed the *R*_g_ distributions (Supplementary Fig. 4b,c), the relative populations of I, Int1, Int2 and A (Fig. 3c), and the distribution of A1/A2 and A2/A3 interdomain distances as a function of free calcium concentration (Fig. 3d–f and Supplementary Fig. 5).

In the presence of EGTA, the *R*_g_ distribution displays a major peak at 2.5 nm, and minor populations with *R*_g_ values broadly distributed ∼4.0–5.5 nm (Supplementary Fig. 4b,c), which correspond to 85–90% inactive conformation, 5% A and 9% Int1, respectively (Fig. 3c,d and Supplementary Fig. 5a). The open conformation Int2 is highly disfavoured with a population of 0–1%, suggesting that the transition to the active conformation following release of A3 is a fast process in these conditions. The presence of the minor populations is apparent in the significant improvement of *χ*_exp_ observed when using an optimized ensemble of 20 models (*χ*_exp_=1.088) rather than a single best-fitting model of the inactive state to represent the data (*χ*_exp_=1.919; Supplementary Fig. 6a). Accordingly, the minimal number of models required for optimal data fitting is constant at around seven models across the different calcium concentrations used, demonstrating that conformational heterogeneity exists regardless of the presence of calcium (Supplementary Fig. 6b).

The addition of 10 μM of free calcium stimulated the exchange process (Fig. 3e and Supplementary Fig. 5b), leading to significant changes in the conformational landscape of A1–A3: the proportion of inactive conformers fell to 50%, whereas Int1 and Int2 rose to ∼15% each, and active conformers increased to nearly 20%. Analysis of A2/A3 interdomain distances reveals a small population of Int1 conformers sampling distances very close to the inactive state as the A1/A3 latch opens up (Fig. 3e and Supplementary Fig. 5b,d), and the overall distribution of A2/A3 distances within the optimized ensemble is markedly different from that of the pool (Supplementary Fig. 5f). It is noteworthy that this significant increase in conformational dynamics and shift to the active conformation happens at a relatively low free calcium-to-protein ratio (0.1:1), which may be due to binding/unbinding of a single calcium ion to A1–A3 occurring much faster than the conformational changes it induces.

At ∼500 μM of free calcium (calcium-to-protein ratio 5:1), which is enough to saturate the three conserved type II calcium-binding sites, the population of active conformers reaches a peak at ∼50%, while the inactive form drops to 20%. Int1 and Int2 represent, respectively, 10 and 20% (Fig. 3c,f). This shift towards a main population of active conformers is apparent in *R*_g_ distributions (Supplementary Fig. 4b,c), with the appearance of a broad peak centred at 3 nm. Interestingly, the selected active conformers preferentially sample relatively short distances between A1 and A2 compared with the pool distribution (similar to the G1–G2 distance in gelsolin-bound actin; Fig. 3f and Supplementary Fig. 5c,d,h), which may reflect a conformational optimization of the A1/A2 linker for actin binding. At even higher free calcium concentrations (2 mM, calcium-to-protein ratio 20:1), the open state (Int2) becomes favoured over the active conformation (55 versus 5%), while the inactive conformation is reduced to 5% (Fig. 3c and Supplementary Fig. 4c). These observations are in line with the increase in the measured *R*_g_ (3.6 nm; Table 2), and the predominance of the open state may explain the degradation of A1–A3 that led to crystallization of A3 in the presence of 5 mM calcium.

### MDS of calcium binding to inactive A1–A3

**Figure 4:**
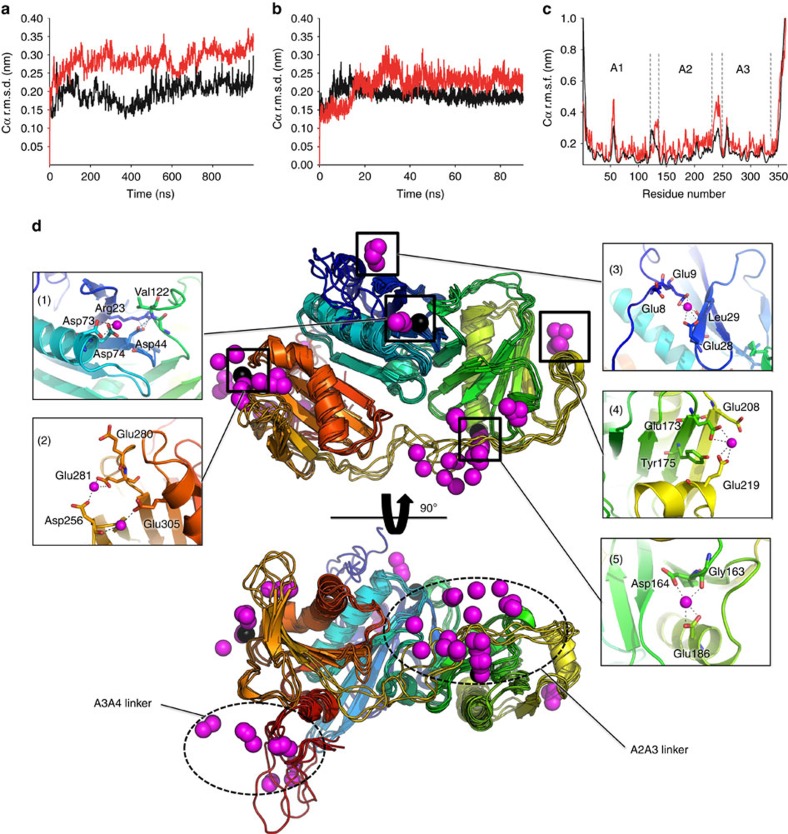
MDS of A1–A3 in the inactive state. (**a**,**b**) r.m.s.d. of Cα atoms from the starting structure in the presence of 50 mM CaCl_2_ (**a**) or NaCl (**b**). The results from two independent simulations are shown as black and red lines, respectively. (**c**) Root mean square fluctuations (r.m.s.f.) of Cα atoms along the protein sequence. The averages of the two independent simulations are shown as black and red lines for systems simulated in the absence and presence of calcium, respectively. (**d**) Analysis of calcium binding to inactive iA1–A3 by MDS. Calcium ions displaying the lowest time-averaged distance to protein atoms were extracted from eight snapshots taken at 100 ns of interval and are represented as magenta spheres. Four overlaid snapshots of the protein are shown in two views rotated by 90° in cartoon representation with the protein backbone coloured from blue to red (N- to C terminus). The protein backbone from four additional snapshots, for which the calcium ions are shown, have been hidden for clarity. Close ups of the calcium-binding sites of interest are shown as insets.

Ensemble optimization demonstrated a complex calcium-induced remodelling of A1–A3 conformational landscape. To gain insights into the initial events that shift the equilibrium towards the active state, we performed explicit solvent MDS of iA1–A3 in the presence of 50 mM CaCl_2_ or 50 mM NaCl (Fig. 4). Although well above the physiological range, such high calcium concentrations enable the detection of regions that display conformational sensitivity to calcium on fast timescales, as well as potential low-affinity sites, within reasonable simulation times (for example, ref. 33). For each system, we ran two independent simulations of 1 μs and 0.1 μs in the presence and absence of calcium, respectively. The protein structure was stable over the course of the simulations, with the r.m.s.d. from the starting structure stabilizing ∼3–4 Å after an initial rise during the first 20 ns (Fig. 4a,b). Analysis of the root mean square fluctuations indicated flexibility mainly in the linker regions, which was enhanced by calcium (Fig. 4c).

We identified bound calcium by analysing time-averaged distances between Ca^2+^ and protein atoms (Fig. 4d). Ca^2+^ clusters mainly around the conserved type II binding sites (Fig. 4d, inset 1, 2 and 5), as well as the A2A3 linker and the C-terminal disordered extension belonging to the A3A4 linker, in a manner that is consistent with the distribution of negative charges on the protein surface (Supplementary Fig. 7). Additional Ca^2+^ is observed between the short α-helices of A1 and A2, and their second and fourth β-strands, respectively (Fig. 4d, inset 3 and 4). These sites involve calcium coordination by Glu9 and Glu28 carboxylates, and the backbone oxygen of Leu29 in A1, and Glu219 and Glu173 carboxylates and the hydroxyl of Tyr175 in A2. These interactions create additional contacts between the short α-helices and β-strands of A1 and A2, and may represent low-affinity-binding sites that stabilize the GH domains at high calcium concentrations.

Examination of Ca^2+^ dynamics at the high-affinity sites indicates that only the type II binding site at A2 can bind Ca^2+^ in the inactive conformation (Fig. 4d, inset 5), showing stable coordination of Ca^2+^ by Asp164 and Glu186 side chains, as well as Gly163 carbonyl oxygen. In contrast, binding at A1 is hindered by the interaction of Asp44 with Arg23 side chain and Val122 backbone nitrogen, resulting from the interaction with A2 (Fig. 4d, inset 1). Similarly, formation of the type II binding site in A3 is prevented by the kink of the long α-helix that interacts with A2, resulting in distances between Glu305, Asp256 and Glu281 that are too large to coordinate calcium tightly (Fig. 4d, inset 2).

**Figure 5:**
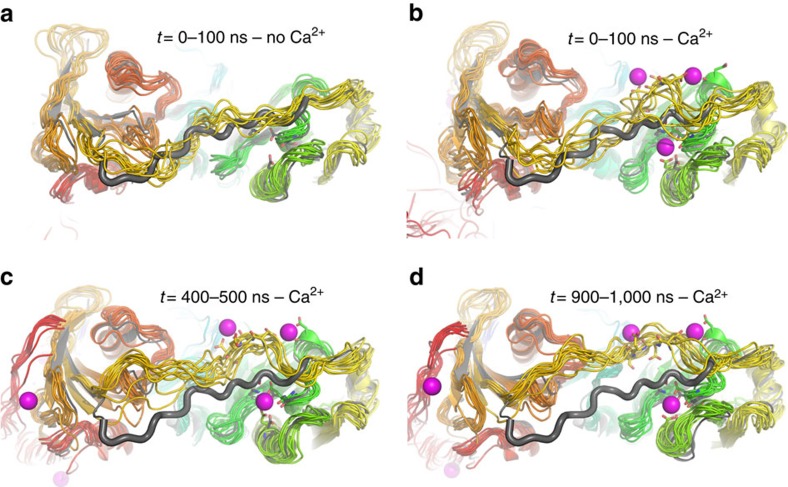
Effect of calcium on A2A3 linker dynamics as observed in MDS. In each panel, the crystal structure of iA1–A3 is shown in grey cartoons for comparison with the A2A3 linker represented using a larger cartoon loop radius. (**a**) Linker dynamics in the absence of calcium. Ten overlaid snapshots of the protein taken at 10 ns of interval (10–100 ns) are shown in cartoon representation with the protein backbone coloured from blue to red (N- to C terminus). (**b**–**d**) Linker dynamics in the presence of calcium, illustrating the increased flexibility and deviation from the crystal structure. Ten overlaid snapshots of the protein taken at 10 ns of interval were extracted from one of the two 1 μs MDS of iA1–A3 in the presence of calcium and are shown as rainbow-coloured cartoons (0–90, 400–490 and 900–990 ns in **b**–**d** respectively). In each case, bound calcium ions are represented in magenta spheres for one of the snapshots, with coordinating side chains as sticks (100 ns in **b**, 500 ns in **c** and 1,000 ns in **d**).

Next, we analysed the effect of calcium on the dynamics of the A2A3 linker, which is located close to the type II binding site at A2 (Fig. 5), and in actin-bound G1–G3 contacts calcium through Asp259 side chain (Asp238 in adseverin), thus providing additional stability to the active state^20^. In the absence of calcium, the extended A2A3 linker samples the conformational space in the vicinity of the crystal structure (Fig. 5a). In contrast, calcium perturbs the linker dynamics, leading to an initial increase in flexibility within the first 100-ns of simulation (Fig. 5b) followed by sampling of calcium-induced, non-native conformational states of the linker associated with calcium binding to the type II binding site in A2 and to the acidic patch of the A2A3 linker (Fig. 5a,d). The observation of calcium-induced changes in A2A3 linker dynamics suggests that this linker acts as a calcium-sensitive conformational switch that modulates the stability of the inactive, intermediate and active states.

### Impact of A1/A3 interface mutations on F-actin severing

**Figure 6:**
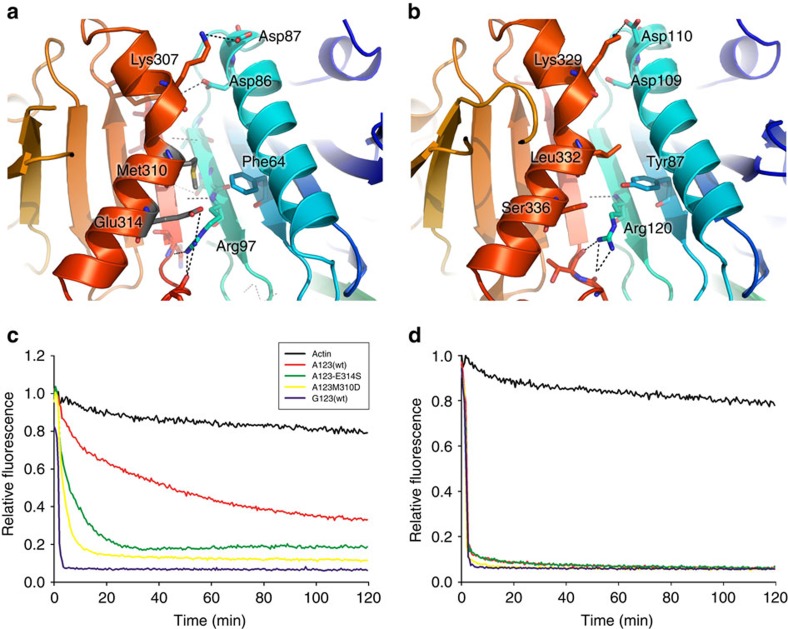
Calcium-independent actin-severing activity of A1–A3 is increased by mutations that disrupt the A1/A3 interface. (**a**) Details of the A1/A3 interface. The mutated residues Met310 and Glu314 are shown as grey sticks. (**b**) Similar view of the G1/G3 interface. (**c**,**d**) Actin depolymerization assays. A total of 6 μM of each protein (actin control, black; A1–A3wt, red; E314S mutant, green; M310D mutant, yellow; and G1–G3, purple) was added to 6 μM of F-actin in the presence of 1 mM EGTA (**c**) or 0.5 mM CaCl_2_ buffer (**d**).

The detailed picture of the Ca^2+^-dependent activation mechanism of A1–A3 brought by the combination of X-ray crystallography, SAXS and MDS explains its reported Ca^2+^-dependent severing activity, which contrasts with the open conformation and Ca^2+^-independent severing of actin filaments described for the equivalent fragment of gelsolin (G1–G3; refs 30, 31). These differences prompted us to investigate the contribution of the stability of the A1/A3 latch to severing activity. To this end, two mutants of A1–A3 (Met310Asp and Glu314Ser) were designed that were aimed at disrupting hydrophobic interactions between Met310 and Phe64, and a salt bridge involving Glu314 and Arg97 at the A1:A3 interface (Fig. 6a). Interestingly, structural comparisons show that in gelsolin, the equivalent residue to Glu314 is a serine, resulting in loss of the salt bridge, while the Met310/Phe64 pair is replaced by Leu332/Tyr87, suggesting a potentially less stable G1:G3 interface (Fig. 6b).

F-actin depolymerization by A1–A3 and its mutants, as well as by G1–G3 was monitored by the loss of fluorescence of pyrene-labelled F-actin in the presence of 1 mM EGTA (Fig. 6c) or 500 μM CaCl_2_ (Fig. 6d). In the presence of Ca^2+^, all proteins showed a similar actin filament depolymerization pattern, with rapid loss of fluorescence. In contrast, in the presence of EGTA, A1–A3 and the M310D and E314S mutants showed reduced severing activity, while only G1–G3 severing activity remained robust, as expected from previous reports^29^,^30^. Interestingly, A1–A3 severing of F-actin was roughly 10 times slower than in the presence of Ca^2+^ (Fig. 6c,d). Although the M310D and E314S mutants showed increased activity compared with wild-type A1–A3, they remained much less active than G1–G3 in Ca^2+^-free conditions, suggesting that the destabilization of the A1/A3 latch in the mutants may only partially shift the equilibrium towards the active state. In addition, M310D was slightly more active than E314S, indicating a larger contribution to destabilizing the interface, as may be expected based on the nature of the mutation (hydrophobic to charged versus charged to hydrophilic).

## Discussion

The structural data presented here have revealed that isolated A1–A3 can adopt, in the crystal and in solution, an inactive conformation (I) characterized by the formation of the A1/A3 latch as observed in calcium-free full-length gelsolin. Using SAXS and ensemble optimization, we have demonstrated that the conformational landscape of A1–A3 is composed of a heterogeneous mixture of dynamically exchanging conformers, where the inactive conformation coexists with activation intermediates and gelsolin-like active conformers in a 9:1 ratio in the absence of calcium. The addition of moderate (10 μM)-to-high (500 μM) calcium concentrations increased conformational exchange, resulting in a larger proportion of intermediate forms (30%), and progressively shifted the equilibrium towards the active form, up to a maximum of ∼55%. These calcium concentrations correlate well with the ∼ 50 μM peaks in subplasmalemmal calcium observed in adrenal chromaffin cells^34^, which enable adseverin to depolymerize the cortical network of actin filaments. Interestingly, we found that higher calcium concentrations (2 mM, close to blood plasma calcium concentration of 1.0–1.5 mM) induced further expansion leading to a main population of open conformers (Int2). Because of its intrinsic disorder, this open conformation may result in faster actin binding and severing through a fly casting mechanism^35^,^36^, and increase the activity of the protein when present in the extracellular environment, for example, following injury as adseverin is highly expressed in soft tissue^3^,^37^. The predominance of Int2 at high calcium concentration may explain the slow degradation of A1–A3 and crystallization of A3 in the presence of 5 mM calcium owing to the increased accessibility of the A1A2 and A2A3 linkers to proteolytic cleavage. This crystal structure revealed the presence of a single calcium ion bound at the type II binding site, associated with the straightened long α-helix that is characteristic of the active forms of gelsolin domains G3 and G6, highlighting the similarity of Ca^2+^-induced conformational changes in both proteins.

**Figure 7:**
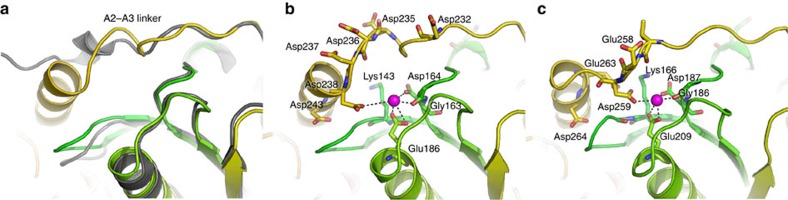
Role of the A2A3 linker in activation and comparison with gelsolin. (**a**) Conformation of the A2A3 linker in the inactive structure (grey cartoons) and active model (rainbow-coloured cartoons) of A1–A3. The crystal structure of iA1–A3 was superimposed onto a homology model of the gelsolin-like active conformation through structural alignment of A2. (**b**,**c**) Calcium coordination at the type II binding site of domain 2 in the active A1–A3 model (**b**) and in the crystal structure of actin-bound G1–G3 (PDB 3FFK) (**c**), highlighting sequence divergence in this region with a more acidic linker in adseverin.

Classical MDS provided detailed insights into the binding of calcium to inactive A1–A3, indicating that binding at A2 can occur in the inactive state while binding at the other type II calcium sites on A1 and A3 is hindered by interdomain contacts (Fig. 4). This bound calcium likely plays a major role in shifting the equilibrium towards the active state at low calcium concentrations by perturbing the conformation of the A2A3 linker (Fig. 5), which undergoes significant refolding during the inactive-to-active transition (Fig. 7a). However, an additional, perhaps more important, mechanism by which calcium binding at A2 may shift the conformational landscape is through stabilization of the active A2:A3 interface (Fig. 7b), likely resulting in an increased lifetime of the active conformation. The inaccessibility of the A1 and A3 type II calcium-binding sites in the inactive conformation implies that the increase in the population of active conformers observed at calcium-to-protein ratios >1 is a consequence of calcium binding at A1 and A3 in the Int1, Int2 and A conformers, which, in combination with the straightening of the long α-helix of A3, prevents the molecule from transitioning back to the inactive form. Finally, we show that very high calcium concentrations destabilize the active A2:A3 interface, leading to a majority of open conformers. Based on the MDS results, we propose that additional calcium binding in the A2A3 linker region, and in particular at the highly acidic stretch ^232^-DGGDDDD-^238^, may cause the observed destabilization. Interestingly, potential additional low-affinity sites that might play a role in stabilizing the A1 and A2 domain structure were also identified in MDS (Fig. 4), although they have not been verified experimentally. The A2A3 linker appears to be optimized for calcium-induced conformational change through allosteric regulation by association with calcium (Fig. 5). Furthermore, ^232^-DGGDDDD-^238^ in the A2A3 linker may offer sequential aspartic acids to the occupied type II calcium-binding site on A2. Thus, as A3 approaches A2, a pathway of calcium/aspartic acid interactions may lead to the docking of the A2:A3 interface, which is stabilized by the final aspartic acid in the sequence (Asp238) coordinating the type II calcium bound to A2 (Fig. 7). Such functionality in linker regions is beginning to be recognized across a range of multidomain proteins^38^.

Actin depolymerization assays have shown that A1–A3 is able to slowly sever actin filaments in EGTA conditions, although this activity was roughly 10% of the severing rate observed in the presence of 500 μM calcium. Interestingly, this correlates well with the proportion of active conformers detected by SAXS in similar conditions, which are, respectively, 5 and 50% in the presence of 2 mM EGTA and 500 μM calcium. This suggests that F-actin binding by A1–A3 is dominated by conformational selection of the active form, and that the inactive-to-active transition may be relatively slow compared to F-actin binding and severing, preventing population shift from occurring. Alternatively, the large concentration of G-actin present in the assay might hinder population shift through sequestering of G-actin by A1–A3 following an initial binding event to the inactive conformation.

The M310D and E314S mutants of A1–A3 showed increased severing activity in the absence of calcium when compared with wild-type A1–A3, most probably due to a partial shift towards the active conformation, which highlighted the importance of the A1/A3 contacts in stabilizing the inactive state. However, the activity of the mutants in calcium-free conditions remained much lower than for wild-type A1–A3 in the presence of calcium, or G1–G3 that displays fast depolymerization activity regardless of the presence of calcium^30^. The fact that these mutations were unable to completely turn A1–A3 into a Ca^2+^-independent severer suggests that the high level of activity of G1–G3 does not primarily result from the instability of the inactive state G1–G3. In support of this, comparison of predicted *Δ*^*i*^*G* for the interfaces between domains 1–2 and 1–3 in iA1–A3 and iG1–G3 using the Pisa Web server^39^ revealed no major difference in stability, and a ∼150-ns MDS of iG1–G3 suggested that the G1:G3 interface is stable on such timescales (Supplementary Fig. 8), also indicating that the domain rearrangements required for activation are slow.

A second structural difference between the two proteins may reside in the stability of the active form, which is tightly influenced by the sequence of the A2A3 linker. Indeed, the concentration of negative charges in the highly acidic stretch ^232^-DGGDDDD-^238^ of A1–A3, as well as the loss of ionic interactions between Lys166 and Glu263, may lead to an active structure that is less stable than aG1–G3 (Fig. 7b,c). Consistent with this, SAXS studies of G1–G3 and fragments G1–G2 and G2–G3 showed that G1–G3 mainly adopts an active/open conformation in solution regardless of calcium concentration, explaining its calcium-independent activity^31^. Although ensemble analysis was not performed on this data, the construct used (gelsolin residues 1–371) has the same length as A1–A3 (residues 1–365+5 residues remaining from the cleaved purification tag), which enables direct comparisons between measured *R*_g_. Interestingly, a mild compaction of G1–G3 from 3.4 to 3.2 nm was observed upon addition of 1 mM calcium. This *R*_g_ of 3.2 nm fits well with the *R*_g_ of A1–A3 in the presence of 500 μM calcium, which is dominated by active conformers. It is thus tempting to speculate that this slight calcium-induced compaction of G1–G3 may result from a shift between open (Int2) and active conformations through stabilization of the G2:G3 interface by calcium binding at the type II site of G2. In addition, the same study showed that mutations in the G2G3 linker could induce Ca^2+^-dependent severing activity in G1–G3. The mutants were found to adopt a collapsed state in the absence of calcium (*R*_g_=2.6 nm similar to inactive A1–A3) and expand in the presence of 1 mM calcium to *R*_g_=3.8 nm. These dimensions are too large to represent exclusively active conformers and suggest the presence of fully expanded conformers, possibly resulting from destabilization of the linker structure. These results, together with the predominance of the open state of A1–A3 at high calcium concentrations, the changes in dynamics associated with the binding of calcium ions to the A2A3 linker observed in MDS, and sequence divergence in this region between adseverin and gelsolin indicate that the linker between domains 2 and 3 plays a critical role in controlling protein dynamics by tuning the relative stability of the inactive, intermediate and active conformations.

In conclusion, we have drawn a quantitative picture of the conformational landscape of A1–A3 and provided structural insights into how calcium binding induces large conformational changes by affecting the relative stability of the accessible conformational states of A1–A3. Our biophysical characterization of A1–A3 in solution helps in explaining the apparent structural similarity yet different calcium-regulated dynamics that characterize the N-terminal halves of adseverin and gelsolin. The origin of these differences is best understood in the context of the full-length proteins. In the absence of a gelsolin C-terminal α-helix latch, adseverin requires tighter interactions than gelsolin in the A1/A3 latch to maintain calcium control of activation.

## Methods

### Cloning, expression and purification

The cloning and *Escherichia coli* expression of the N-terminal halves of adseverin (A1–A3, residues 1–365) and gelsolin (G1–G3, residues 25–372) have been described previously in refs 23, 20. Briefly, the constructs were engineered into a modified pET-21d(+) (Novagen) plasmid, pSY5, to encode an 8-histidine tag, followed by a PreScission protease cleavage site, ahead of the N terminus of the proteins. The A1–A3 M310D and E314S mutants were generated using the QuikChange site-directed mutagenesis kit (Stratagene) and the following oligonucleotide pairs: 5′- CCCAAGAGAGGAAGGCTGCAGATAAGACAGCTGAAGAATTTC -3′; 5′- GAAATTCTTCAGCTGTCTTATCTGCAGCCTTCCTCTCTTGGG -3′ for M310D and 5′- GGCTGCAATGAAGACAGCTTCAGAATTTCTACAGCAAATG -3′; 5′ CATTTGCTGTAGAAATTCTGAAGCTGTCTTCATTGCAGCC -3′ for E314S. Constructs were transformed into *E. coli* Rosetta 2 (DE3) cells (Novagen) for expression according to the manufacturer's protocol. Overnight starter cultures were prepared by growing the cells in LB liquid media supplemented with 100 μg ml^−1^ ampicillin and 34 μg ml^−1^ chloramphenicol at 37 °C with shaking at 250 r.p.m. for 16 h. Fresh LB liquid media for expression of each construct was inoculated with 50 ml of overnight starter culture per liter, and incubated with shaking at 250 r.p.m. at 37 °C until cell density reached OD_600_=0.6. Proteins were expressed by induction with 1 mM isopropyl β-D-1-thiogalactopyranoside at 23 °C overnight. Cells were harvested by centrifugation at 3,700*g* for 30 min. The pellets were subsequently resuspended in 100 ml binding buffer (50 mM Tris-HCl (pH 8.0), 500 mM, NaCl and 20 mM imidazole), and disrupted by 2 mg ml^−1^ lysozyme treatment for 1 h at 4 °C followed by sonication.The suspensions were clarified by centrifugation at 32,000*g* for 45 min in an SS-34 rotor in a RC 5C Plus centrifuge (Sorvall) at 4 °C. Nickel-nitrilotriacetic acid (Ni-NTA) beads (2 ml) were added to the supernatant and mixed continuously for 30 min at 4 °C. The beads were pelleted at 1,000*g* at 4 °C for 15 min, and resuspended in buffer containing 300 mM NaCl, 20 mM imidazole, 10 mM Tris-HCl (pH 7.5). After washing the beads, proteins were eluted by raising the imidazole concentration to 250 mM. The His-tag was cleaved by addition of human rhinovirus 3C protease followed by overnight dialysis against 150 mM NaCl and 20 mM Tris-HCl (pH 7.5). Proteins were further purified by size-exclusion chromatography on a HiLoad 16/60 Superdex 200 prep grade column (GE Healthcare), first equilibrated with 125 ml of 100 mM EGTA, 20 mM Tris-HCl (pH 7.2), 150 mM NaCl, re-equilibrated with 250 ml gel-filtration buffer (0.2 mM EGTA, 20 mM Tris-HCl (pH 7.2) and 150 mM NaCl). Proteins were concentrated to 10 mg ml^−1^ by Ultra-15, MWCO 10 kDa, Amicon Ultra centrifugal filter units.

### Crystallization and data collection

Crystallization was carried out by vapour diffusion using a Cartesian Technologies pipetting system^40^. Solutions containing 10 mg ml^−1^ of A1–A3 in 50 mM NaCl and 10 mM Tris-HCl (pH 7.5) supplemented with 5 mM of either EDTA or CaCl_2_ crystallized in 20% polyethylene glycol 3,350, 100 mM Bis-Tris propane (pH 7.5) or 30% polyethylene glycol 6,000, 100 mM HEPES (pH 7.0), respectively, which led to the crystal structures of iA1–A3 and aA3. Crystals were frozen in liquid nitrogen after being soaked in a mother liquor solution supplemented with 25% glycerol. Diffraction data were recorded on the ID29 and ID14-2 beamlines at the European Synchrotron Radiation Facility, Grenoble, France. Wavelength was 1.033 and 0.933 Å for iA1–A3 and aA3, respectively. All data were automatically processed by xia2 (ref. 41).

### Structure determination and refinement

Calcium-free iA1–A3 and calcium-bound aA3 structures were solved by molecular replacement using the calcium-free N-terminal half of gelsolin, iG1–G3, excised from PDB 1D0N (ref. 26) or the calcium-bound gelsolin domain 3, aG3 excised from PDB 1RGI (ref. 18) as search models in PHASER^42^. The molecular replacement solutions were subjected to repetitive rounds of restrained refinement in PHENIX^43^ and Autobuster^44^, and manual building in COOT^45^. Translation/Libration/Screw (TLS) parameters were included in the final round of refinement. The CCP4 programme suite^46^ was used for coordinate manipulations. The structures were validated with Molprobity^47^. 97 and 99% of residues were in the Ramachandran-favoured region for iA1–A3 and aA3, respectively. Refinement statistics are given in Table 1 and a portion of the electron density map for each structure is shown in Supplementary Fig. 9.

### SAXS experiments

Protein samples (10 mg ml^−1^ in 0.2 mM EGTA, 20 mM Tris-HCl (pH 7.2) and 150 mM NaCl) were mixed with 10 × calcium or EGTA stock solutions to achieve the required free calcium concentrations, which were calculated using the Ca-EGTA Calculator v1.3 webserver (http://maxchelator.stanford.edu/CaEGTA-TS.htm)^48^. SAXS data were collected at the European Synchrotron Radiation Facility on beamline ID14-3. The sample-to-detector distance was 1 m and the wavelength of the X-rays was 0.0995, nm. Data acquisition was performed at 20 °C. Protein concentrations ranged from 1 to 8 mg ml^−1^. Data reduction was performed using the established procedure available at ID14-3, and buffer background runs were subtracted from sample runs. The radius of gyration was determined with the programme PRIMUS^49^ according to the Guinier approximation at low *Q* values, in a *Q.R*_*g*_ range up to 1.3:


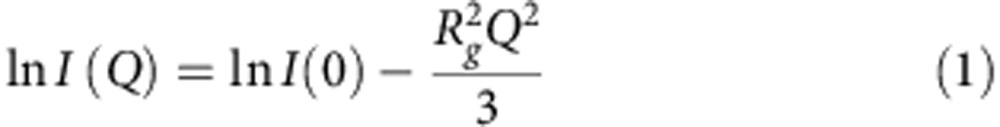


The pairwise distance distribution functions, *P(r)*, were calculated with the programme GNOM^50^. Molecular weights were estimated based on ref. 51.

### Ensemble generation

An ensemble approach was used to model the N-terminal half of adseverin (A1–A3). We employed all-atom coarse-grained MDS^52^ to generate candidate structures based on available X-ray crystallographic data. Starting coordinates for the inactive state (I) were obtained from the crystal structure of calcium-free N-terminal half of adseverin or from a homology model based on PDB 1RGI for the active structure (A)^18^. Complete models including the missing disordered termini were generated in Modeller^53^. An additional homology model based on the structure of the gain-of-function mutant of the actin capping protein CapG (PDB 2J72; ref. 54), which differs from the active structure by having a different interface between domains 2 and 3, was also generated and used as a qualitative control of the accuracy of the ensemble optimization approach. Starting conformers for intermediates 1 and 2 (Int1 and Int2) were obtained by dissociating domain 3 and then domain 1 from the iA1–A3 model. This was performed by defining and altering backbone torsions within the interdomain linker regions in VEGA ZZ^55^. An additional model for Int2 was generated by dissociating domain 3 from domain 2 in the active structure, leading to an alternative conformer of Int2 where the alpha helix in the A2A3 linker is folded. Models of A1–A3 (I, Int1, Int2, A and the CapG-derived model) were simulated in GROMACS^56^ using an atomistic coarse-grained structure-based model^57^,^58^. A timestep of 0.0005 time units was used and the simulation was coupled to a temperature bath via Langevin dynamics. 10,000 Snapshots were extracted from each simulation, and a 1,000 models from each simulation were randomly dispatched into 10 independent ensembles of 6,000 models.

### Ensemble optimization

For each model from each ensemble, theoretical SAXS patterns were calculated with the programme CRYSOL^59^, and ensemble optimization fitting was performed for each of the 10 independent ensembles with GAJOE^60^. GAJOE uses a genetic algorithm to select from a large pool of conformers optimized sub-ensembles that minimize the discrepancy between the experimental and calculated curves *χ*_exp_ according to the following equation:


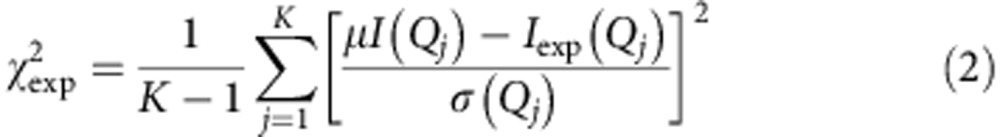


where *K* is the number of points in the experimental curve, *σ* is the s.d. and *μ* is a scaling factor. The ensemble size was varied from 1 to 50 models and variations in the quality of the fit to the experimental data *χ*_exp_ were monitored in order to analyse protein flexibility (Supplementary Fig. 6). Additional validation of the ensemble calculations was also performed by fitting the experimental data to pool ensembles including only I, A or I+A models, which yielded fits of poorer quality than when using ensembles that included the intermediate states (I+Int1+Int2+A; Supplementary Table 1 and Supplementary Fig. 3). In order to build conformational landscapes, 50 models were selected per ensemble, resulting in 50 × 10=500 optimized models. Interdomain distances were extracted from the models using GROMACS routines and then used to calculate two-dimensional histograms (using a binning of 15 × 15). The relative populations of conformers were counted in each optimized ensemble and averaged for each experimental SAXS profile to gain insight into the mechanism of calcium activation.

### Explicit solvent MDS

Classical MD simulations of iA1–A3 in the presence and absence of calcium were performed using GROMACS 4 (ref. 56) and the AMBER99SB-ILDN force field^61^. At the beginning of each simulation, the protein was immersed in a box of SPC/E water, with a minimum distance of 1.0 nm between protein atoms and the edges of the box. An amount of 50 mM of either CaCl_2_ or NaCl were then added using genion. Long-range electrostatics were treated with the particle-mesh Ewald summation^62^. Bond lengths were constrained using the P-LINCS algorithm. The integration timestep was 5 fs. The v-rescale thermostat and the Parrinello–Rahman barostat were used to maintain a temperature of 300 K and a pressure of 1 a.t.m. Each system was energy minimized using 1,000 steps of steepest descent and equilibrated for 200 ps with restrained protein heavy atoms. For each system, two independent production simulations were obtained by using different initial velocities. The aggregated simulation time was 200 and 2,000 ns for the simulations in the absence and presence of calcium, respectively. r.m.s.d. and root mean square fluctuations were calculated using GROMACS routines.

### Actin depolymerization assays

Polymerization buffer (final concentration, 50 mM KCl, 0.2 mM ATP, 2 mM MgCl_2_, 0.5 mM dithiothreitol, 1 mM EGTA and 50 mM HEPES, pH 7.5) was added to 10% pyrene-labelled G-actin (6 μM) in 96-well, flat-bottomed plates (Corning), and was incubated for 30 min to allow the formation of F-actin. Calcium was then added to obtain the required free calcium concentration. Reactions were equilibrated for 1 h before 6 μM of the respective proteins (A1–A3, A1–A3-M310D, A1–A3-E314S or G1–G3) were added. The final volume in each well was 100 μl. Fluorescence intensity was measured kinetically at excitation and emission wavelengths of 365 and 407 nm, respectively, using a Safire2 fluorimeter (Tecan).

## Additional information

**How to cite this article:** Chumnarnsilpa, S. *et al*. Calcium-controlled conformational choreography in the N-terminal half of adseverin. *Nat. Commun.* 6:8254 doi: 10.1038/ncomms9254 (2015).

## Supplementary Material

Supplementary Information Supplementary Figures 1-9, Supplementary Table 1, Supplementary Note 1 and Supplementary References.

## Figures and Tables

**Table 1:** Data collection and refinement statistics for iA1–A3 and aA3.

	**iA1**–**A3 (PDB ID: 5A1K)**	**aA3 (PDB ID: 5A1M)**
*Data collection*		
Space group	C2	I422
Cell dimensions		
*a, b, c* (Å)	135.4 , 65.3, 105.2	92.5, 92.5, 86.9
*α, β, γ* (°)	90, 118.3, 90	90, 90, 90
Resolution (Å)	59.6–2.9 (3.0–2.9)	35.5–1.8 (1.9–1.8)
*R*_merge_, %	15.3 (133.0)	10.8 (88.9)
Average *I*/*I*_o_	10.4 (1.5)	21.6 (3.5)
Completeness, %	98 (95)	100 (97)
Redundancy	5.5 (5.5)	14.2 (14.1)
		
*Refinement*		
Resolution (Å)	59.6–2.9 (3.0–2.9)	35.5–1.8 (1.9–1.8)
No. of reflections	17,745 (2,818)	13,831 (2,615)
*R*_work_/*R*_free_	24.0 (30.2)/24.8 (33.3)	18.2 (20.4)/18.9 (22.5)
*No. of atoms*		
Protein	5,402	815
Ligand/ion	0	1
Water	107	58
*B-factors*		
Protein	61	20
Ligand/ion	0	17
Water	51	28
r.m.s.d.		
Bond lengths (Å)	0.008	0.008
Bond angles (°)	1.120	0.980

r.m.s.d., root mean squared deviation.

**Table 2:** SAXS-derived parameters.

**EGTA/free calcium concentration**	**c (mg ml**^−1^)	**MW (kDa)**	*R*_g_ **(nm)**	**D**_**max**_ **(nm)**	**χ**_**exp**_
10 mM EGTA	1.00	40	2.64	9.0	0.915
10 mM EGTA	2.00	42	2.62	9.1	1.123
10 mM EGTA	4.00	39	2.65	9.1	1.524
10 mM EGTA	8.00	40	2.67	9.3	1.674
2 mM EGTA	1.00	39	2.61	9.1	0.832
2 mM EGTA	2.00	39	2.61	9.3	1.098
2 mM EGTA	4.00	39	2.69	9.1	1.198
2 mM EGTA	8.00	40	2.70	9.2	1.461
10 μM Ca^2+^	1.00	36	3.05	10.0	0.803
10 μM Ca^2+^	2.00	39	3.05	10.6	0.865
10 μM Ca^2+^	4.00	38	2.95	10.3	0.939
10 μM Ca^2+^	8.00	39	2.90	10.1	1.291
220 μM Ca^2+^	1.00	33	3.07	10.4	0.786
220 μM Ca^2+^	2.00	37	3.02	10.4	0.862
220 μM Ca^2+^	4.00	38	2.96	10.3	0.947
220 μM Ca^2+^	8.00	40	2.98	10.4	1.361
380 μM Ca^2+^	1.00	36	3.03	10.5	0.827
380 μM Ca^2+^	2.00	36	3.03	11.5	0.862
380 μM Ca^2+^	4.00	38	3.02	10.0	1.162
380 μM Ca^2+^	8.00	40	2.97	9.9	1.281
490 μM Ca^2+^	1.00	35	3.10	10.5	0.830
490 μM Ca^2+^	2.00	36	3.10	10.4	0.874
490 μM Ca^2+^	4.00	39	3.19	10.2	0.914
490 μM Ca^2+^	8.00	40	3.19	10.5	1.131
2 mM EGTA	4.00	38	2.56	8.8	1.386*
19 nM Ca^2+^	—	38	2.57	8.9	1.323*
49 nM Ca^2+^	—	38	2.60	8.7	1.343*
175 μM Ca^2+^	—	38	2.91	9.7	1.006*
550 μM Ca^2+^	—	39	3.16	10.9	0.947*
2.3 mM Ca^2+^	—	43	3.61	12.3	1.262*
50 mM Ca^2+^	—	43	3.67	13.5	2.053*

SAXS, small-angle X-ray scattering.

^*^Optimal fitting of the data was obtained using ensembles that include a deletion of the last 15 C-terminal amino acids (residues 350–365) that belong to the A3–A4 linker and are intrinsically disordered in solution.
