# Supplementary material for: Calcium-controlled conformational choreography in the N-terminal half of adseverin
Source: Nat Commun. 2015 Sep 14;6:8254. doi: 10.1038/ncomms9254 (PMC4647846; doi:10.1038/ncomms9254)
Supplement: Supplementary Information — Supplementary Figures 1-9, Supplementary Table 1, Supplementary Note 1 and Supplementary References. [file ncomms9254-s1.pdf]

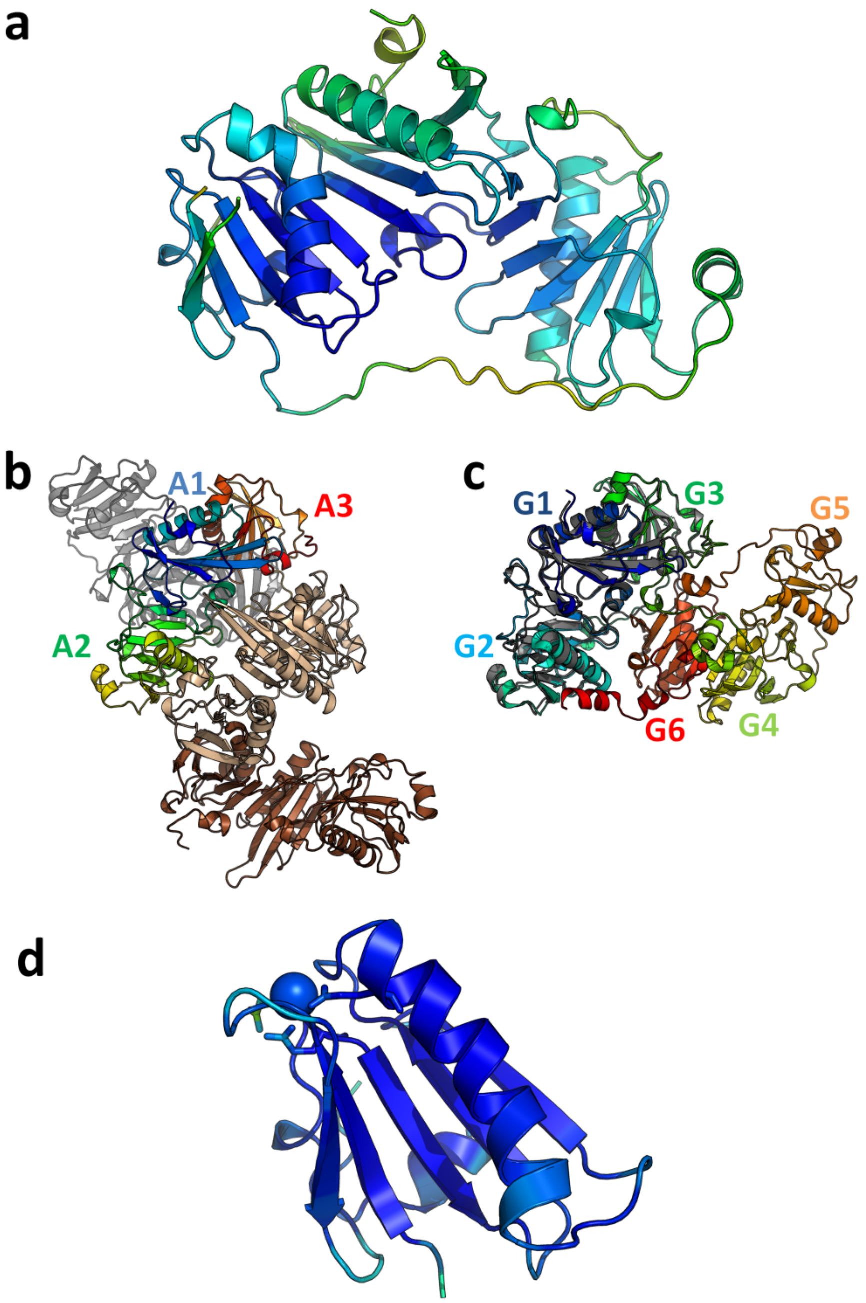

Supplementary Figure 1: Crystallographic packing and flexibility of iA1-A3 and aA3 in their crystalline states. a. B-factor representation of inactive A1-A3. b. inactive A1-A3 molecules pack through a relatively large interface that involves contacts with all three domains. c. Comparison of iA1-A3 with the structure of full-length gelsolin (PDB 1D0N), showing that the crystallographic packing interface of iA1-A3 largely covers the surfaces that make contacts with the C-terminal half in the full-length protein. Here iA1-A3 is in grey and in the same orientation as shown in b. d. B-factor representation of calcium bound A3.

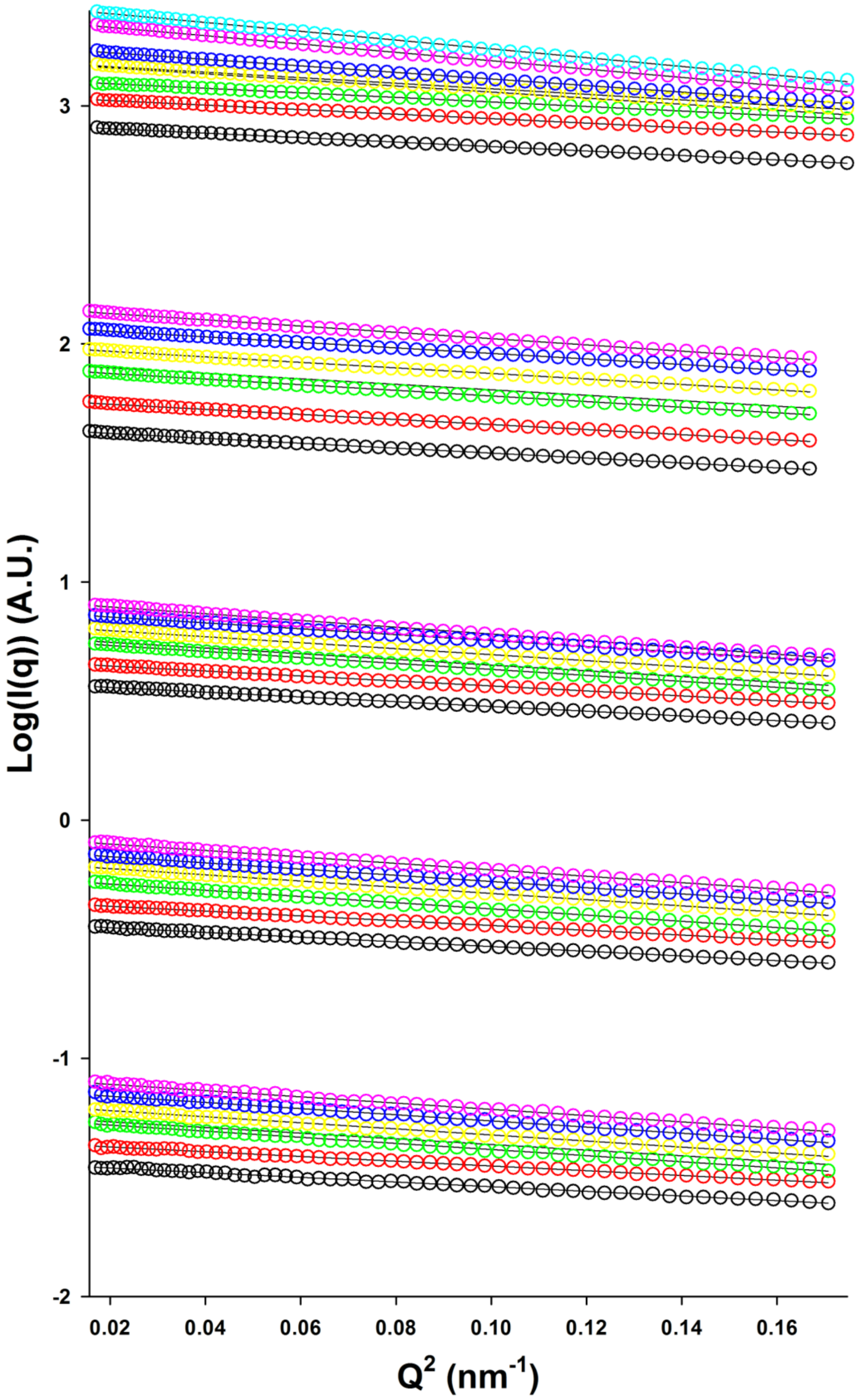

Supplementary Figure 2: Guinier representations (Log(I(Q)) vs Q^2) of the data shown in figure 2a, showing linearity in the low Q region.

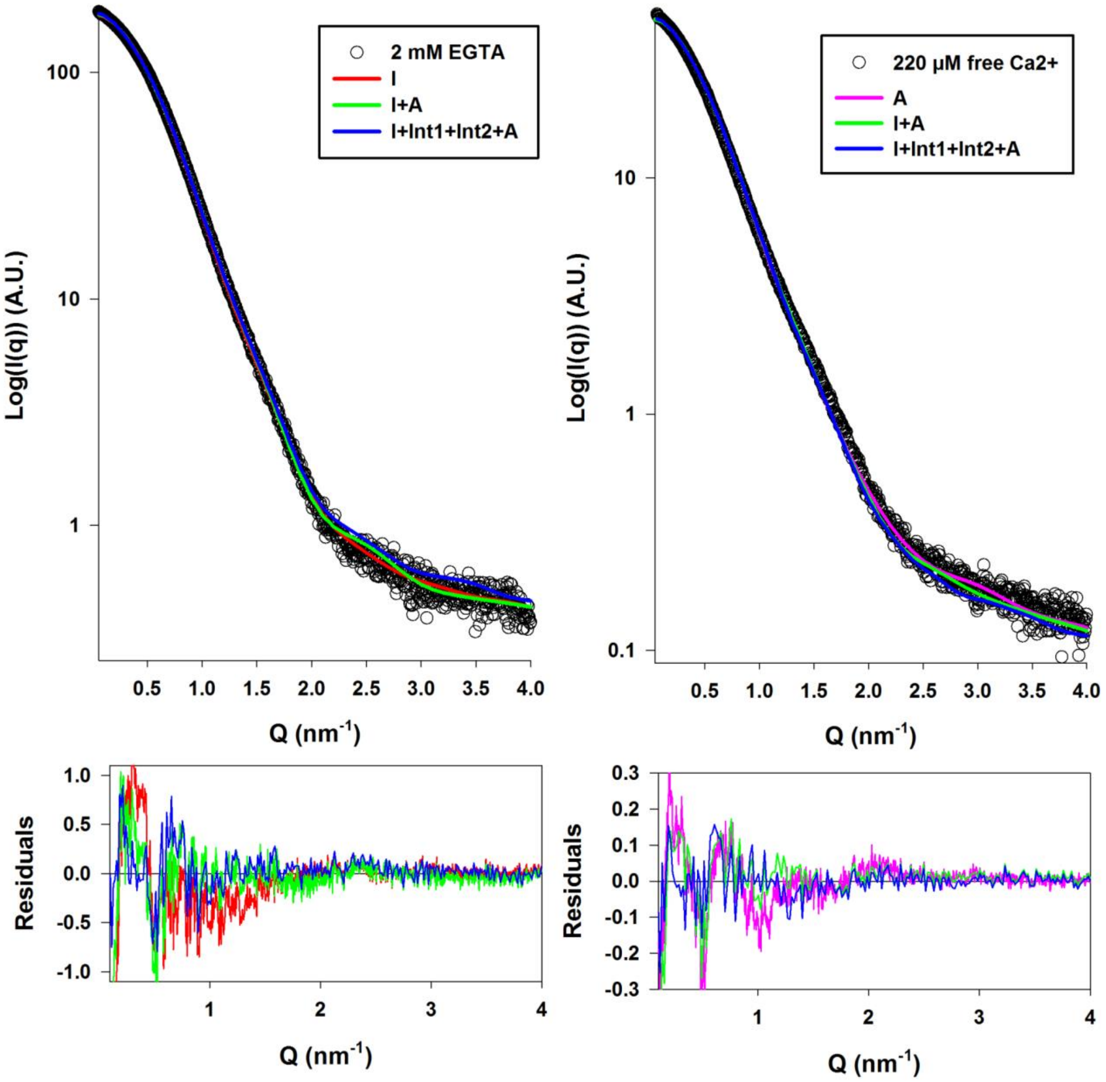

Supplementary Figure 3: fitted SAXS profiles of data measured at 8 mg.ml^{-1} in the presence of 2 mM EGTA (a) or 220 µM free calcium (b) using different pool ensembles. χ_{exp} values are shown in Supplementary table 1. (a) data was fitted using EOM version 2 using a pool of inactive (I), inactive and active (I+A), or inactive, intermediate states and active states (I+Int1+Int2+A). (b) data was fitted using EOM version 2 using a pool of active (A), inactive and active (I+A), or inactive, intermediate states and active states (I+Int1+Int2+A). (c) and (d). Residuals of the fits shown in (a) and (b).

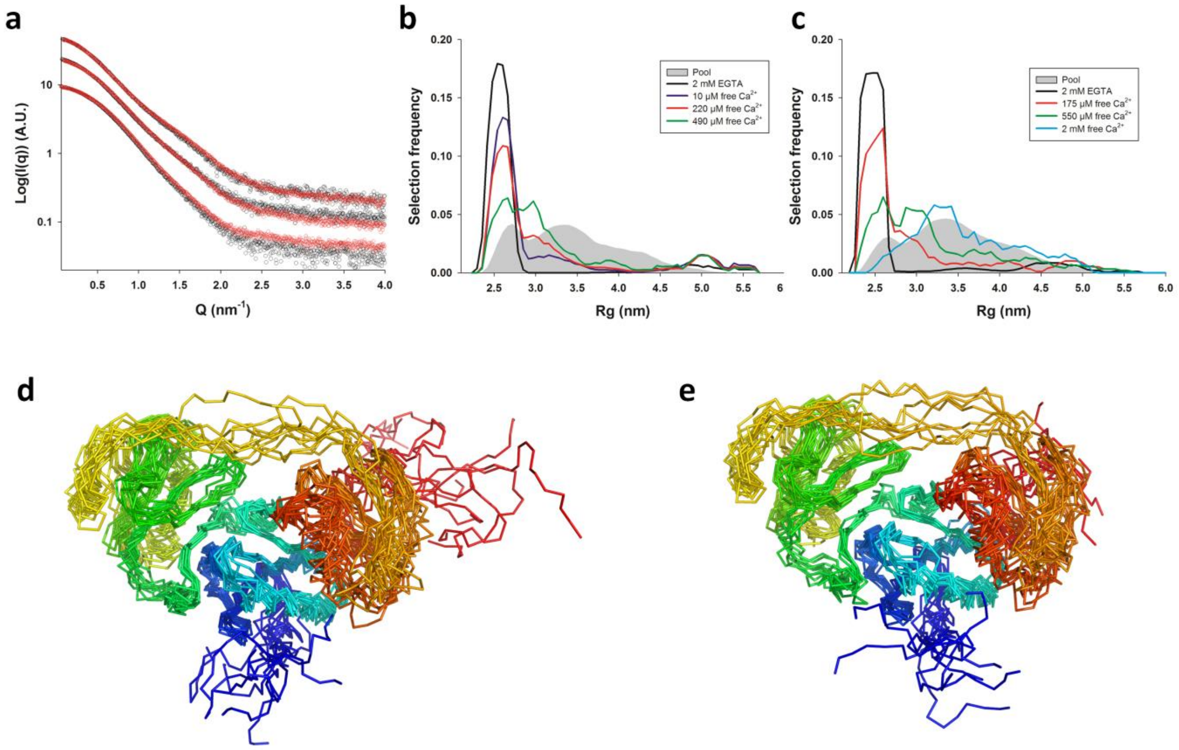

Supplementary Figure 4: comparison of the two SAXS datasets measured using different protein preparations and influence of degradation of the last 15 C-terminal residues on the analysis. (a) comparison of SAXS data measured at 4 $mg.ml^{-1}$ during two different synchrotron visits (old data in black, new data in red). From bottom to top, the conditions are 2 mM EGTA, 175 µM (old) or 220 µM (new) free calcium, and 490 µM (old) or 550 µM (new). Note the improvement in signal-to-noise ratio in the most recent dataset, which was due to technical improvements at the beamline. (b) and (c). Radius of gyration distributions obtained through ensemble optimization using old (b) or new (c) data. The pool ensemble used in (c) included models with a deletion of the last 15 C-terminal amino acids. Both datasets yielded similar Rg distributions in the absence of calcium (black lines) and when exposed to comparable levels of free calcium (red and green lines) (d) and (e). Comparisons of inactive state models extracted from optimized ensembles fitted against data measured in the presence of 2 mM EGTA using old (d) or new (e) data. 10 overlaid models are shown in ribbon representation and colored from blue to red (N- to C-terminus), highlighting the deletion of the disordered C-terminal tail.

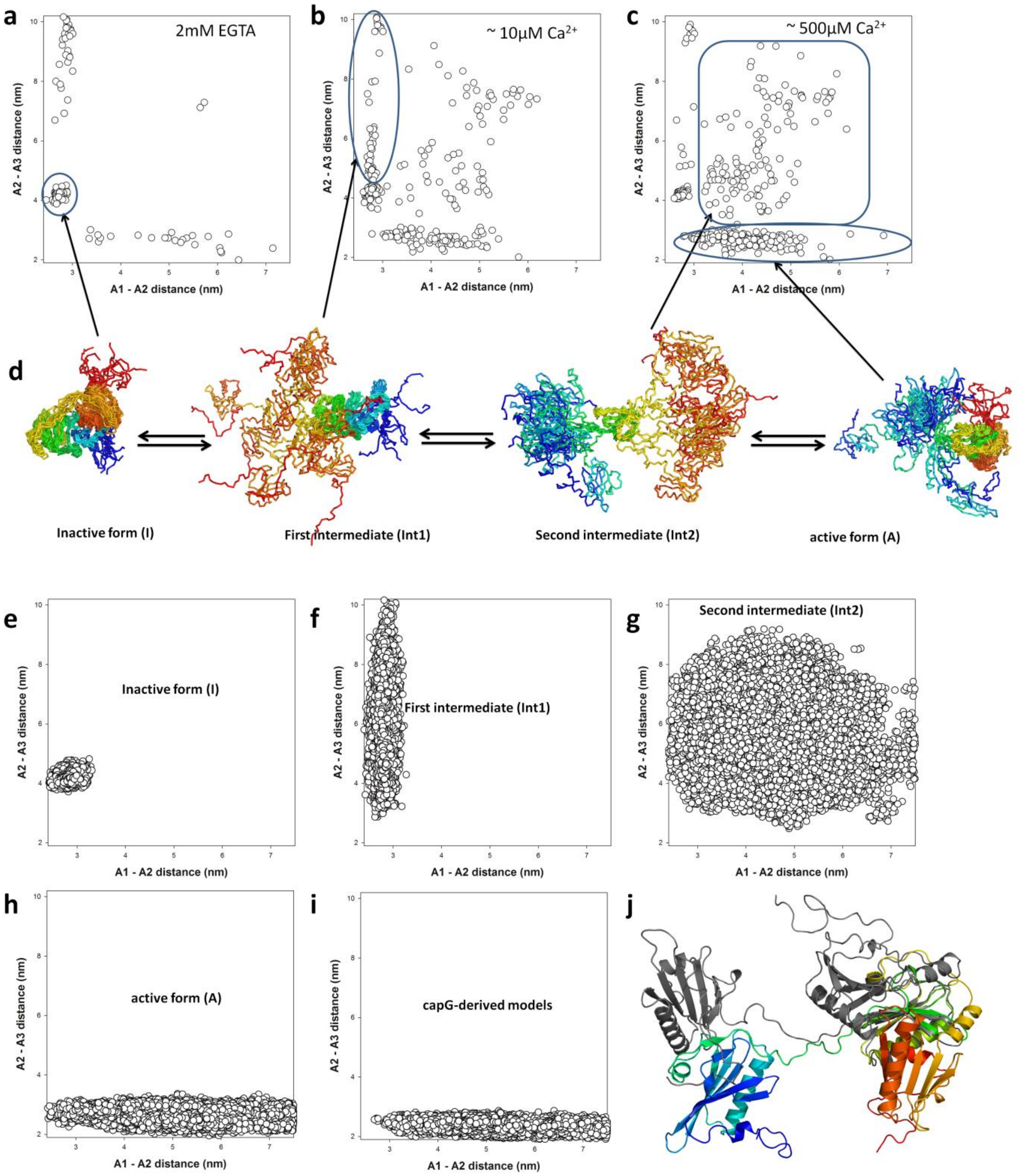

Supplementary Figure 5: structural features of the optimized ensembles. (a), (b) and (c). The distributions of interdomain distances (A1-A2 versus A2-A3) for the models of the optimized ensembles of 500 models shown in figure 3d,e and f are represented as scatter plots in the presence of 2 mM EGTA (a), 10 µM free calcium (b) or 500 µM free calcium (c). (d) inactive-to-active transition of A1-A3. For each conformational state, 10 superimposed models extracted from optimized ensembles of 50 models are shown in red and coloured from blue to red (N-to C-terminus), highlighting the variations in interdomain distances and orientations. (e), (f), (g), (h) and (i) scatterplot representations of the distributions of A1-A2 and A2-A3 interdomain distances for the

pool ensembles of inactive (e), intermediate 1 (f), intermediate 2 (g), active state (h) and capG-derived models (i). (j) comparison of the active (rainbow coloured) and capG-derived models (gray). Two models are shown in cartoon representation and aligned onto domain two, highlighting the different interface between domain two and three.

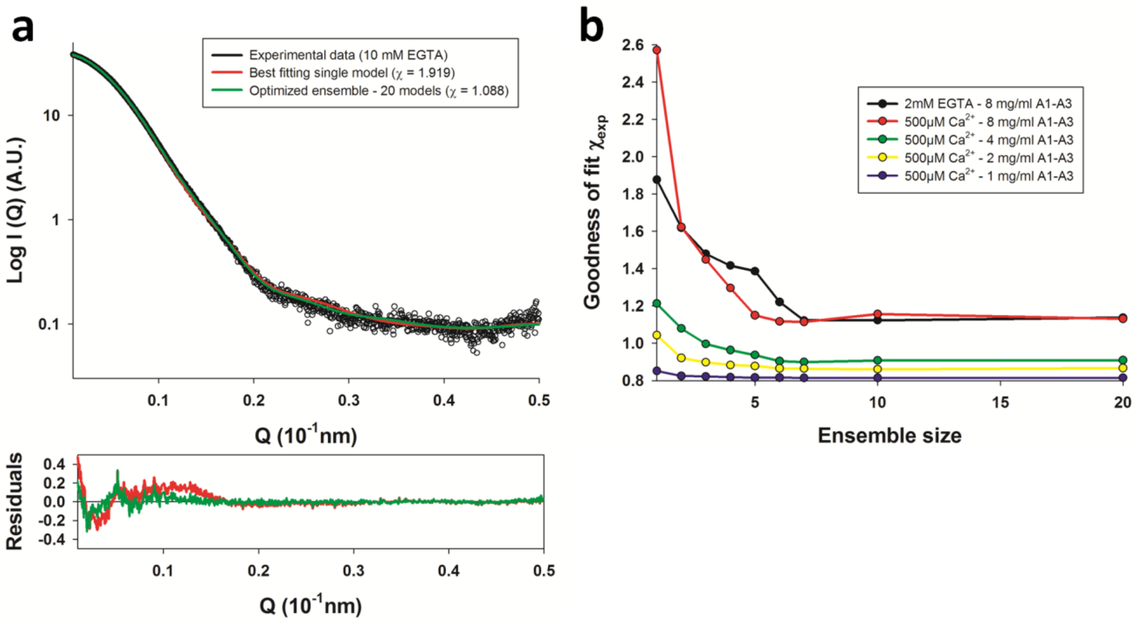

Supplementary Figure 6: Variations of the goodness of fit to the experimental SAXS data χ_{exp} with the number of models present in the optimized ensemble. (a). Top: Improvement of the quality of the fit with an ensemble of 20 models (green line) compared to a single model of inactive A1-A3 (red line), for data measured in the presence of 10 mM EGTA. The experimental curve is shown in black. Bottom: residuals of the fits, showing the small but significant improvement in the goodness of fit (b). Changes in the quality of the fit as a function of optimized ensemble size.

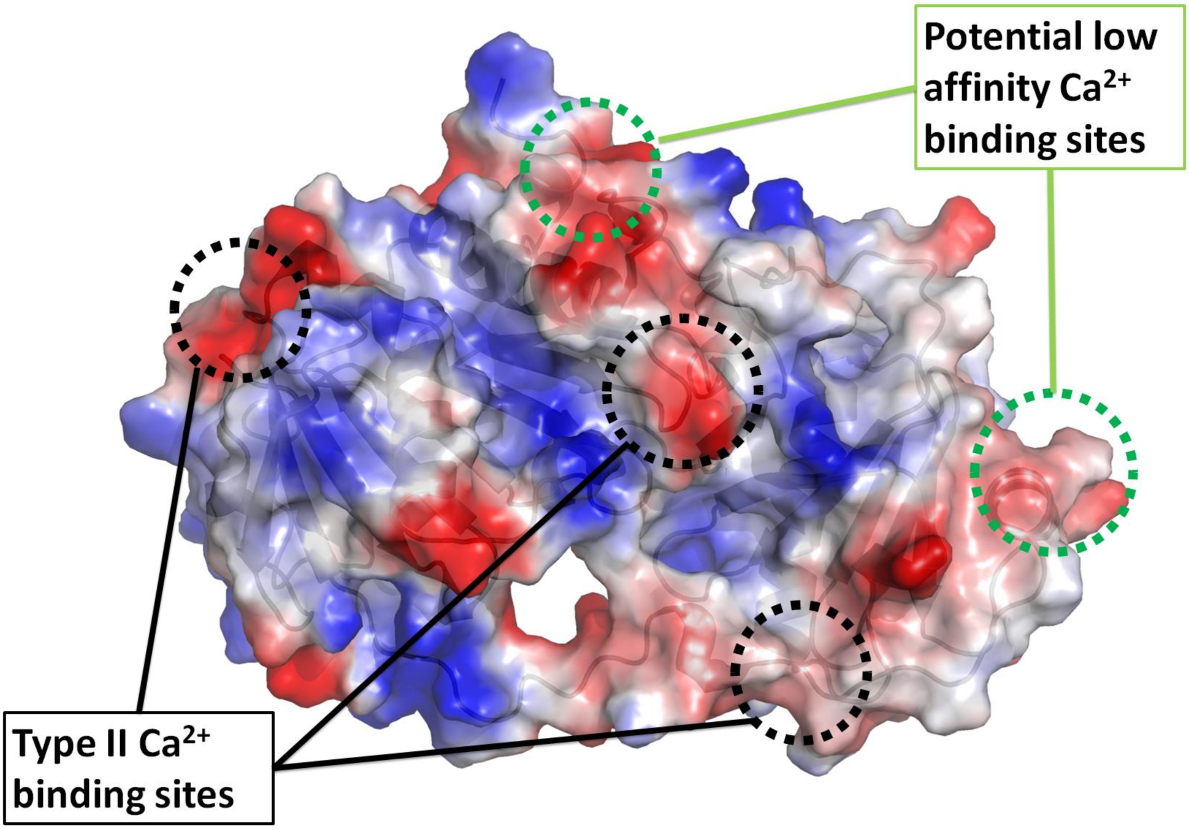

Supplementary Figure 7: Distribution of charged residues on the surface of inactive iA1-A3. The *e*lectrostatic surface, drawn using vacuum charges in PyMOL, is shown with partial transparency and overlaid on a cartoon representation. The regions where bound calcium ions were observed in molecular dynamics simulations are indicated by dotted circles.

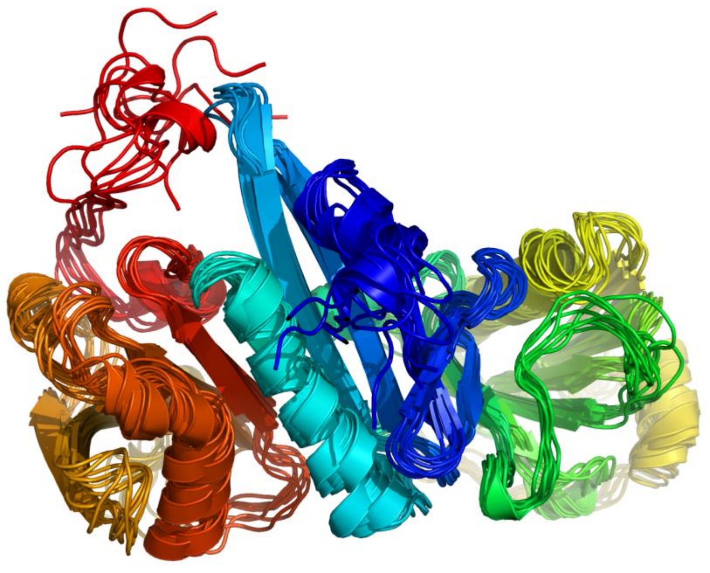

Supplementary Figure 8: Snapshots of inactive G1-G3 taken at 20 ns interval and extracted from a 150 ns classical, explicit-solvent molecular dynamics simulation. The protein is shown in cartoon representation and coloured from blue to red (N- to C-terminus).

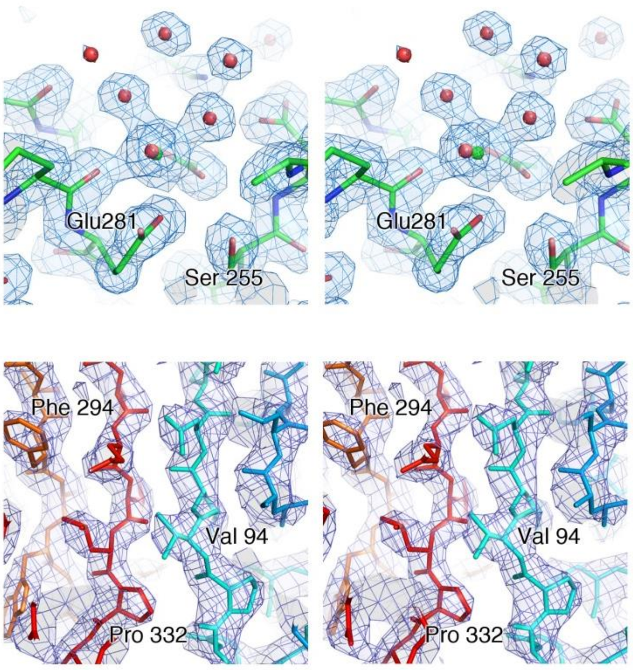

Supplementary Figure 9: Stereo image showing a portion of the 2*Fo-Fc* electron density map for the aA3 (top panel) and iA1-A3 (bottom panel) crystal structures. The maps were contoured at 1.5σ.

[A1-A3] = 8 mg.ml^{-1}	Optimized ensemble χ_{exp}* (0.08 < Q < 5 nm^{-1})					
EGTA/Free Ca^{2+} concentration	Inactive	Active	Inactive + Active	Inactive + Intermediates + Active	Inactive + Intermediates + Active + capG-derived models**	Random***
10 mM EGTA	2.79	21.3	1.28	1.13	1.13	49.6
2 mM EGTA	2.98	18.5	1.58	1.25	1.25	49.0
10 µM Ca^{2+}	10.9	2.27	1.61	1.12	1.12	37.7
220 µM Ca^{2+}	9.52	2.64	1.60	1.06	1.06	44.0
380 µM Ca^{2+}	8.50	2.24	1.45	1.08	1.08	35.0
490 µM Ca^{2+}	16.75	1.86	1.69	1.16	1.16	29.1

Supplementary Table 1: Quality of the goodness of fit χ_{exp} using different pool ensembles.

* Calculations were performed using model-weighting and automated optimization of ensemble size, as implemented in EOM version 2 [1].
** no change was observed upon inclusion of capG-derived models as they were not selected from the pool.
*** The pool was generated in Ranch using sequence information and the "Compact" option for model generation. High resolution X-ray structures were not used .

Supplementary Note 1: Rationale for the modeling of A1-A3 conformational landscape and validation of ensemble calculations.

The Ca^{2+}-induced changes in the scattering profile, together with available X-ray crystallographic data and homology with gelsolin, suggest that the inactive-to-active transition involves large-scale conformational changes of A1-A3, as shown in figure 3a. The release of the A1/A3 latch from the inactive form (I) leads to a first intermediate (Int1), followed by the loss of the A1:A2 interface (Int2). Finally, the Ca^{2+}-stabilized A2:A3 interface is formed, resulting in a gelsolin-like active conformation (A). Although the instability of the Int1 and Int2 forms has so far prevented their detection by structural methods, their existence has been postulated on the basis of the apparent topological changes between inactive gelsolin and active G1-G3 bound to actin [2,3]. SAXS is ideally suited to report on these conformational changes in solution, although the analysis is complicated in the case of A1-A3 by the presence of similarly folded globular domains that can interact through distinct interfaces, leading to potential ambiguity in the discrimination of different forms with similar shapes and flexibility (for example, Int1 and A). For this reason, previous studies on gelsolin and fragments have been limited to *ab initio* shape reconstructions [4,5], which carry significant bias due to conformational averaging and the lack of treatment of molecular flexibility [6,7].

In order to overcome these limitations and reach a more quantitative description of the A1-A3 conformational landscape, we used atomistic coarse-grained MDS to sample the available conformational space of I, Int1, Int2 and A, providing a large collection of physically accessible states that can be used to retrieve the underlying populations present in the observed experimental scattering profiles through ensemble optimization. We extensively validated our ensemble calculations by systematically fitting the experimental data with pool ensembles that sample exclusively the I or A states, or combinations of pools (I + A or I+ Int1 + Int2 + A), demonstrating a small but significant improvement in the quality of the fit when modeling in intermediate states (Supplementary table 1 and Supplementary Figure 3). We additionally included in the calculations an ensemble of models derived from the structure of Eu^{3+}-bound mutant human macrophage capping protein CapG (PDB 1JHW) [8], which differs from the gelsolin-like active form by having a different interface between domains 2 and 3 (Supplementary Figure 5j), in order to assess whether the information content of the SAXS data is sufficient to discriminate between these relatively similar models (ensemble I+ Int1 + Int2 + A + capG - Supplementary table 1). All models were combined in large pools and sub-ensembles that described the experimental SAXS profiles were selected using ensemble optimization (see methods and Supplementary table 1).

The selection frequency of the CapG-derived models lay between 0 and 2% in all ensemble calculations. This indicates that the information content of the SAXS data is sufficient to filter out an incorrect A2/A3 interface from the optimized ensembles models while having distributions of A1-A2 distances similar to the active state (Supplementary Figure 5 h, i and j).

Supplementary references

1. Tria, G., Mertens, H.D., Kachala, M. & Svergun, D.I. Advanced ensemble modelling of flexible macromolecules using X-ray solution scattering. *IUCrJ* **2**, 207-17 (2015).
2. Burtnick, L.D., Urosev, D., Irobi, E., Narayan, K. & Robinson, R.C. Structure of the N-terminal half of gelsolin bound to actin: roles in severing, apoptosis and FAF. *EMBO J* **23**, 2713-22 (2004).
3. Nag, S. et al. Ca2+ binding by domain 2 plays a critical role in the activation and stabilization of gelsolin. *Proc Natl Acad Sci U S A* **106**, 13713-8 (2009).
4. Ashish et al. Global structure changes associated with Ca2+ activation of full-length human plasma gelsolin. *J Biol Chem* **282**, 25884-92 (2007).
5. Peddada, N. et al. Global shapes of F-actin depolymerization-competent minimal gelsolins: insight into the role of g2-g3 linker in pH/Ca2+ insensitivity of the first half. *J Biol Chem* **288**, 28266-82 (2013).
6. Bernado, P. & Svergun, D.I. Analysis of intrinsically disordered proteins by small-angle X-ray scattering. *Methods Mol Biol* **896**, 107-22 (2012).

7. Receveur-Brechot, V. & Durand, D. How random are intrinsically disordered proteins? A small angle scattering perspective. *Curr Protein Pept Sci* **13**, 55-75 (2011).
8. Zhang, Y. et al. A CapG gain-of-function mutant reveals critical structural and functional determinants for actin filament severing. *EMBO J* **25**, 4458-67 (2006).
